# A Case of Infiltrative Cardiomyopathy Secondary to Primary Hyperoxaluria Type 2 - Utilization of Multimodality Imaging

**DOI:** 10.7759/cureus.17914

**Published:** 2021-09-12

**Authors:** Dae Hyun Lee, Thomas Kasprowicz, Alberto Morales, Ignacio Sotolongo, Joel Fernandez, Ravi Korabathina

**Affiliations:** 1 Cardiology, University of South Florida, Tampa, USA; 2 Cardiology, South Tampa Cardiology, Tampa, USA; 3 Cardiology, Bayfront Health Medical Center, St. Petersburg, USA

**Keywords:** primary hyperoxaluria, cardiomyopathy, infiltrative cardiomyopathy, cardiac mri, oxalate

## Abstract

Primary hyperoxaluria is a rare genetic disorder characterized by oxalate crystal deposition, including in the heart. Hyperoxaluria-associated cardiomyopathy manifests as restrictive infiltrative cardiomyopathy. We present a case of a 52-year-old male with a past medical history of type 2 primary hyperoxaluria, end-stage renal disease on hemodialysis, paroxysmal atrial fibrillation, and hypertension, who presented with dyspnea and lethargy. Transthoracic echocardiogram showed cardiomyopathy with ejection fraction (EF) of 35-40% with severe hypokinesis of apical myocardium. Endomyocardial biopsy revealed interstitial fibrosis and crystal deposition consistent with oxalate. Cardiac MRI showed late gadolinium enhancement with subendocardial, nearly transmural fibrosis of lateral wall along with mid myocardial involvement of anterior and septal wall. To the best of our knowledge, this is the first case of type 2 primary hyperoxaluria-associated cardiomyopathy utilizing transthoracic echo, endomyocardial biopsy, and cardiac MRI.

## Introduction

Primary hyperoxaluria is a rare genetic metabolic disorder that can be divided into two subcategories: primary hyperoxaluria type 1 (PH type 1, more common) and primary hyperoxaluria type 2 (PH type 2, less common) [1].

PH Type 2 is due to reduced activity of the glyoxylate reductase enzyme, which is responsible for removing cytosolic glyoxylate, reducing oxalate production. This enzyme is found in the kidneys and is highly concentrated in the liver. Therefore PH type 2 can be diagnosed by measuring the glyoxylate reductase activity in liver biopsy samples [2]. It commonly manifests as urolithiasis and eventual kidney damage from oxalate deposition [3]. Later on, oxalate deposition occurs in the heart - specifically in the conduction system, intramyocardial vessels, and myocytes [4,5]. It causes conduction abnormalities and cardiomyopathy, the major cause of death in these patients [6].

We describe a case of a patient with PH Type 2 who developed hyperoxaluria-mediated cardiomyopathy diagnosed through multi-modality imaging including transthoracic echocardiogram, cardiac MRI, and confirmed with endomyocardial biopsy.

## Case presentation

**Figure 1:**
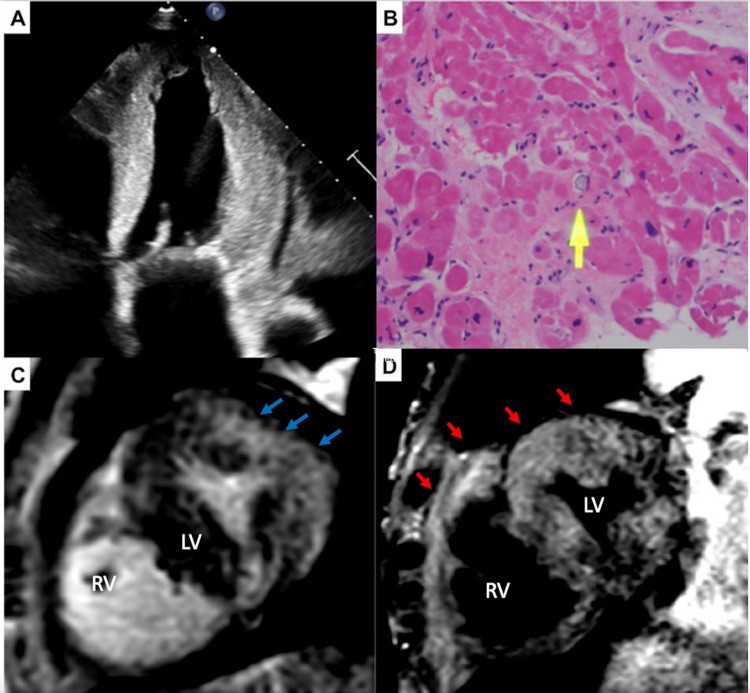
Clinical images for primary hyperoxaluria induced cardiomyopathy Figure 1A: Representative image of the apical four-chamber view in transthoracic echocardiogram shows concentric hypertrophy. Figure 1B: Hematoxylin and eosin (H&E) staining with oxalate deposition (yellow arrow) in the endomyocardial biopsy. Figure 1C: Cardiac MRI images with delayed gadolinium enhancement sequence of short-axis view at the mid-papillary segment showing diffuse subendocardial fibrosis with a transmural pattern along the lateral wall (blue arrows). Figure 1D: Cardiac MRI images with T2-weighted sequence of short-axis view at the mid-papillary segment showing diffuse increased signal intensity in the myocardium of both right and left ventricle (red arrows). RV - right ventricle; LV - left ventricle.

A 52-year-old male with comorbidities of type 2 primary hyperoxaluria, liver cirrhosis, end-stage renal disease on hemodialysis, secondary hyperparathyroidism, paroxysmal atrial fibrillation, and hypertension presented to us with dyspnea, lethargy, and lightheadedness during hemodialysis. The patient’s clinical manifestation was significant for acute heart failure with dyspnea. Initial workup including transthoracic echocardiography (TTE) showed reduced left ventricular ejection fraction (EF) of 35-40%, grade 2 diastolic dysfunction, severe left ventricular hypertrophy, severe hypokinesis of apical and moderate hypokinesis of basal anteroseptal segments, severely calcified aortic valve annulus with moderate stenosis, moderately calcified mitral valve annulus with mild stenosis (Figure 1). Due to concern for restrictive/infiltrative cardiomyopathy, further workup, including abdominal fat pad biopsy, was negative for amyloidosis. The patient underwent a right heart catheterization (RHC) with an endomyocardial biopsy.

**Figure 2:**
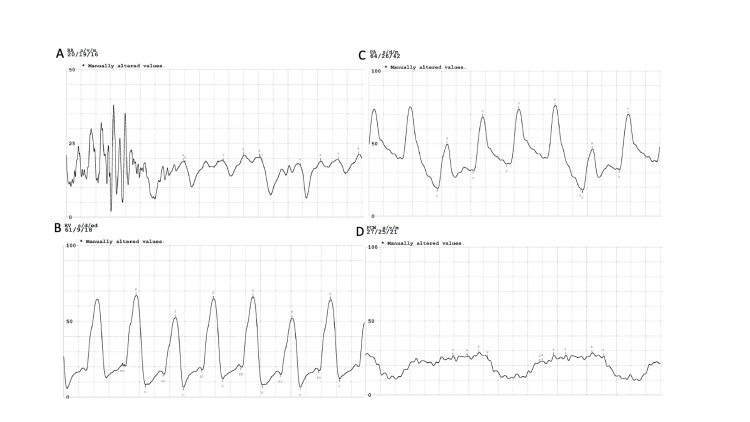
Right heart catheterization hemodynamic tracing Hemodynamic tracing for elevated right atrial pressure (19mmHg, Figure 2A), right ventricular pressure (61/9 mmHg, Figure 2B), pulmonary artery pressure (64/26mmHg, Figure 2C) and pulmonary capillary wedge pressure (21mmHg, Figure 2D)

Right heart catheterization was significant for elevated right atrial pressure (19mmHg), right ventricular pressure (60/23mmHg), pulmonary artery pressure (64/26mmHg, mean pressure 42mmHg), pulmonary capillary wedge pressure (21mmHg; Figure 2). Cardiac output was preserved with 4.7L/m with a cardiac index of 2.5L/min*m^2^. The catheter waveform did not show any constrictive hemodynamics. Endomyocardial biopsy revealed interstitial fibrosis and crystal deposition consistent with oxalate cardiomyopathy (Figure 1).

Cardiac MRI was performed to assess the extent of cardiac involvement. Cardiac MRI demonstrated a reduced left ventricular (LV) ejection fraction of 35%. Delayed gadolinium enhancement showed diffuse subendocardial and transmural fibrosis in the lateral wall of LV and mid-myocardial involvement of anterior and septal walls consistent with an infiltrative process (Figure 1). The T2-weighted sequence showed a diffusely increased signal intensity of the myocardium involving both the right and left ventricle (Figure 1).

Unfortunately, the patient continued to deteriorate clinically and went to hospice care before he passed away.

## Discussion

Our case describes primary hyperoxaluria type 2 induced cardiomyopathy who underwent multimodality imaging, including TTE and cardiac MRI. Although both primary hyperoxaluria subtypes are rare, PH type 2 is the less common subtype. In fact, Wachter et al. discussed the first case of cardiomyopathy secondary to PH type 2. A 41-year-old man with right-sided heart failure on dialysis-dependent renal insufficiency was found to have a reduced left ventricular systolic function and an echo-dense myocardium on an echocardiogram [7]. Since then, several case reports of PH type 2 have been reported [1]. In a comprehensive case series of PH type 2, there was no cardiac manifestation [8].

Despite the difference in the pathogenesis of subtypes of PH, the results are similar - deposition of oxalate crystals in the cardiac tissue causing different manifestations, including conduction abnormality or cardiomyopathy. In a comprehensive analysis of the PH registry in Japan, 31 out of 38 patients had EKG, or echocardiographic abnormalities, most common of which were increased left ventricular mass (42%), left atrial size (31%), pulmonary hypertension (23%), and diastolic dysfunction (15%). Cardiomyopathy (EF <50%) was present in 11% of patients [6]. 

The TTE can be characterized by a “sparkling high-intensity echocardiographic pattern,” which can be due to the increased deposition of oxalate (and calcium) crystals [9]. However, it can be difficult to differentiate between amyloidosis and hyperoxaluria- induced cardiomyopathy- as they both have thickened myocardium, sparkling high intensity of myocardium, and presence of pericardial effusion. Cardiac MRI is a powerful diagnostic tool to evaluate infiltrative cardiomyopathy [10]. Our patient’s MRI results showed late gadolinium enhancement in the sub-endocardial and mid-myocardial walls.

The treatment options are limited, including kidney, or combined kidney-liver transplantation [4]. Kidney transplantation only has a high recurrence rate, whereas combined kidney-liver transplantation reverses enzymatic defect, thus longer survival. Combined liver-kidney transplant allows reversal of cardiomyopathy. Other supportive measures include preserving kidney function and preventing nephrolithiasis with adequate fluid intake and oral citrate supplementation. In addition, dialysis would allow the removal of excess fluid and uremic toxins, including oxalate crystals.

## Conclusions

In conclusion, PH type 2 is a rare genetic disorder that can involve the heart. To the best of our knowledge, we present the first case of PH 2-associated cardiomyopathy utilizing cardiac MRI, which can be an excellent non-invasive diagnostic tool. 
